# Enrichment of Large-Diameter Semiconducting Single-Walled Carbon Nanotubes by Conjugated Polymer-Assisted Separation

**DOI:** 10.3390/nano13132001

**Published:** 2023-07-04

**Authors:** Piao Xie, Yun Sun, Chao Chen, Shu-Yu Guo, Yiming Zhao, Xinyu Jiao, Peng-Xiang Hou, Chang Liu, Hui-Ming Cheng

**Affiliations:** 1 Shenyang National Laboratory for Materials Science, Institute of Metal Research, Chinese Academy of Sciences, Shenyang 110016, China; pxie17s@imr.ac.cn (P.X.); yunsun@imr.ac.cn (Y.S.); chchen19s@imr.ac.cn (C.C.); syguo14s@imr.ac.cn (S.-Y.G.); ymzhao19b@imr.ac.cn (Y.Z.); xyjiao18s@imr.ac.cn (X.J.); cheng@imr.ac.cn (H.-M.C.); 2 Faculty of Materials Science and Engineering/Institute of Technology for Carbon Neutrality, Shenzhen Institute of Advanced Technology, Chinese Academy of Sciences, Shenzhen 518055, China

**Keywords:** semiconducting single-walled carbon nanotubes, disperse, thin-film transistors

## Abstract

Semiconducting single-walled carbon nanotubes (s-SWCNTs) with large diameters are highly desired in the construction of high performance optoelectronic devices. However, it is difficult to selectively prepare large-diameter s-SWCNTs since their structure and chemical stability are quite similar with their metallic counterparts. In this work, we use SWCNTs with large diameter as a raw material, conjugated polymer of regioregular poly-(3-dodecylthiophene) (rr-P3DDT) with long side chain as a wrapping agent to selectively separate large-diameter s-SWCNTs. It is found that s-SWCNTs with a diameter of ~1.9 nm are effectively enriched, which shows a clean surface. By using the sorted s-SWCNTs as a channel material, we constructed thin-film transistors showing charge-carrier mobilities higher than 10 cm^2^ V^−1^ s^−1^ and on/off ratios higher than 10^3^.

## 1. Introduction

Single-walled carbon nanotubes (SWCNTs) have been regarded as an ideal material for use in electronics and flexible optoelectronics due to their excellent optical and electrical properties, including high carrier mobility [1], tunable band gaps [2], and high carrier density [3]. Semiconducting SWCNTs (s-SWCNTs) are an ideal channel material for the construction of flexible transistors [4], photovoltaics [5], photodectors [6], and flexible electronics [7]. However, a slight change in the diameter and/or chiral angle may lead to the transition of SWCNTs from semiconducting to metallic or vice versa. In general, SWCNTs are a mixture of ~1/3 metallic SWCNTs (m-SWCNTs) and ~2/3 s-SWCNTs. The m-SWCNTs would connect D-S electrodes, which forms a permeation path and decrease the on/off ratio of electronic devices. In consequence, obtaining high-purity s-SWCNTs is highly desired in the construction of high performance devices as a channel material. In recent years, a great deal of effort has been devoted to obtain high purity s-SWCNTs, and great progress has been made. One way is to directly grow s-SWCNTs by controlling catalyst composition/structure [8,9,10] or preferential etching [11,12] originating from the intrinsic properties of the chemical activity of m-SWCNT [13,14]. Although enriched s-SWCNTs were previously obtained using the direct-growth method, the s-SWCNT content in the sample and their reproducibility need further improvement. The other dominant way is a post-treatment method by chemical and/or physical treatments of SWCNTs, which has advantages of higher purity and better controllability and repeatability.

By now, many post-synthesis separation techniques, including density gradient centrifugation [15,16], aqueous two-phase extraction [17,18], gel chromatography [19,20], conjugated polymer-assisted separation [21], dielectrophoresis [22], and DNA wrapping [23] have been reported. Among these approaches, conjugated polymer-assisted separation shows great prospects in practical application due to its advantages in selectivity, separation yield and treatment process [24]. Notable progress has been made by using this method, especially in obtaining small-diameter s-SWCNTs [25,26]. The diameters of obtained s-SWCNTs are generally smaller than 1.2 nm. However, s-SWCNTs with diameters larger than 1.2 nm are recently required for optoelectronic devices, due to the higher carrier mobility [27], small Schottky barrier [28], and superior optical characteristics. Various conjugated polymers, including polyfluorene [29], polycarbazole [30], polythiophene [31], and linear D-A-based polymer [32], have been shown to be effective in selectively separating s-SWCNTs. However, the diameter of obtained s-SWCNTs are mostly smaller than 1.5 nm. Additionally, there has been no report in obtaining s-SWCNTs with even larger diameters due to the smaller difference of electronic structure and polarization between such s-SWCNTs and their metallic counterparts [33].

In this study, we use a conjugated polymer of regioregular poly-(3-dodecylthiophene) (rr-P3DDT) with long side chain and extensive π-conjugated structure [31] to selectively disperse large-diameter SWCNTs (mean diameter > 1.7 nm). Through optimizing the separation conditions, s-SWCNTs with a relatively large diameter of 1.9 nm were first obtained. Then, we constructed thin-film transistors (TFTs) using the obtained s-SWCNTs and investigated their performance.

## 2. Experimental Procedure

### 2.1. Synthesis of Large-Diameter SWCNTs

Large-diameter SWCNTs were synthesized in our lab with a common floating catalyst chemical vapor deposition (FCCVD) method using hydrogen as carrier gas. The detailed synthesis conditions, including carbon source and catalyst precursor, were described in our previous reports [34,35].

### 2.2. Enrichment of Large-Diameter s-SWCNTs

The schematic showing s-SWCNT separation process is in Figure S1. Briefly, as-prepared SWCNTs by FCCVD were first magnetically stirred in H_2_O_2_ solution for 24 h at room temperature to introduce oxygen functional groups (Figure S2) in order to disperse the SWCNTs well. Then, the H_2_O_2_-treated SWCNT solution was in turn filtered, rinsed with de-ionized water for multi-cycles, and dried in an oven at 120 °C for 5 h. Rr-P3DDT (purchased from Sigma-Aldrich, Shanghai, China) with a weight of 7.5 mg was dissolved in 15 mL toluene. An amount of 3.75 mg H_2_O_2_-treated SWCNTs was then added into the above toluene solution. Finally, the mixture solution was in turn subjected to ultrasonication for 5 min, tip-ultrasonicated for 30 min, centrifuged for 60 min at 30,000× *g*, and centrifuged for 120 min at 40,000× *g*. The obtained supernatant was denoted as s-SWCNTs-rr-P3DDT.

## 3. Results and Discussion

### 3.1. Characterization of Large-Diameter SWCNT Raw Material

**Figure 1:**
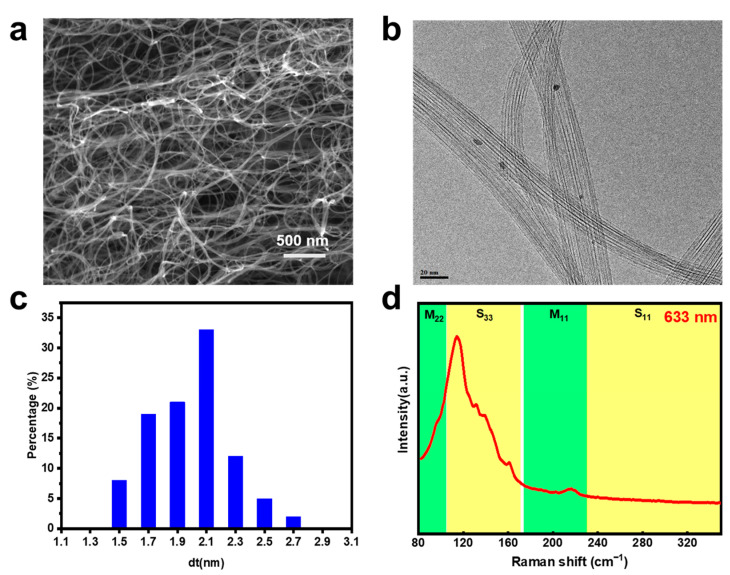
(**a**) SEM image, (**b**) TEM image, (**c**) histogram of diameter, and (**d**) Raman spectra (excited with 633 nm laser) of the as-prepared SWCNTs.

Scanning electron microscopy (SEM) image of the used SWCNT sample is shown in Figure 1a, which demonstrates an interconnected SWCNT network. A typical transmission electron microscope (TEM) image shown in Figure 1b indicates that the network is composed of SWCNT bundles. Observations of 150 bundles suggest that most of the bundle diameter (~65%) is larger than 10 nm (Figure S3). The measured diameter distributions (Figure 1c) of SWCNTs (~200 isolated tubes) under TEM are in the range of 1.5~2.7 nm. Radial breathing mode (RBM) Raman spectra excited from the metallic and semiconducting ones are highlighted in the multi-wavelength Raman spectra shown in Figure 1d and Figure S4 [36], which indicates that m- and s-SWCNTs coexist in the raw sample. We further estimated the raw SWCNT diameter distribution according to the equation based on the relationship of RBM frequency (ω) and SWCNT diameter (d) (ω = 218.3/d + 15.9) [37], which is calculated to be 1.5~2.7 nm, and the same range was maintained with that from the TEM observation. Furthermore, the SWCNT have a high I_G_/I_D_ intensity ratio (~170), suggesting its high quality (Figure S5). The thermogravimetric curve shown in Figure S6 further identified the high quality of our SWCNT raw material.

### 3.2. Separation of s-SWCNTs

**Figure 2:**
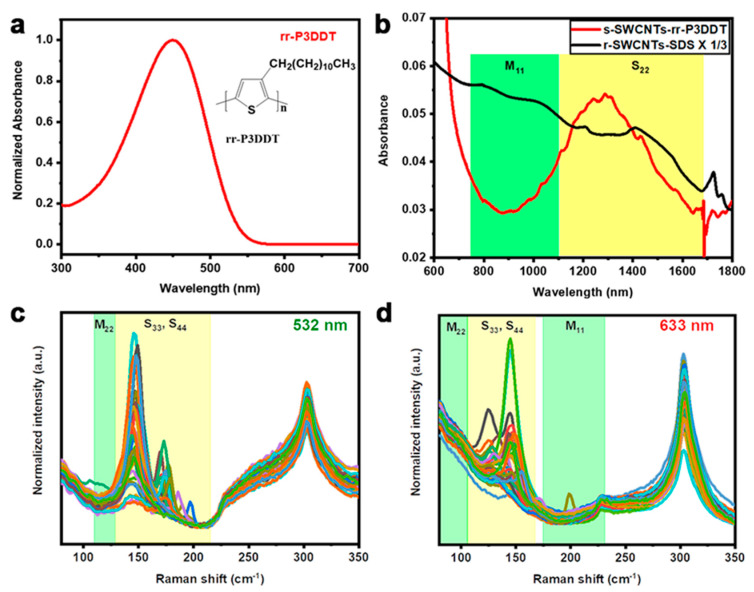
(**a**) UV–Vis–NIR absorbance spectrum of conjugated polymer rr-P3DDT and its chemical structure, (**b**) UV–Vis–NIR absorbance spectra of s-SWCNTs-rr-P3DDT and r-SWCNTs-SDS, (**c**) and (**d**) RBM Raman spectra of s-SWCNTs-rr-P3DDT excited by 532 nm and 633 nm lasers, respectively.

For comparison, we also separated SWCNTs using sodium dodecyl sulfate (SDS) surfactant, which is a commonly used CNT dispersion surfactant, and the obtained supernatant was named as r-SWCNTs-SDS. The samples of s-SWCNTs-rr-P3DDT and r-SWCNTs-SDS were analyzed using ultraviolet–visible–near-infrared (UV-vis-NIR) absorption spectroscopy. The background interference from the absorption of rr-P3DDT is below 650 nm (Figure 2a), which can be neglected, and the background interference from the absorption of SDS (Figure S7) also has very little effect on the absorption peak of s-SWCNTs. The typical UV–vis–NIR spectra of r-SWCNTs-SDS and s-SWCNTs/rr-P3DDT are shown in Figure 2b. Broad absorption peaks ranging from 800 to 1600 nm are detected in both metallic and semiconducting zones for the r-SWCNT-SDS sample identifying the coexistence of metallic and semiconducting tubes. On the contrary, the s-SWCNTs-rr-P3DDT sample shows strong semiconducting peak (S_22_) intensity located in 1120–1600 nm, while the peaks originated from metallic tubes (M_11_) in 800–1100 nm disappeared, suggesting the shortage of m-SWCNTs in the sample. The ϕ value calculated from Figure 2b for the s-SWCNTs-rr-P3DDT is 0.34, suggesting that the s-SWCNT ratio in the solution is higher than 97.5% [38,39]. In addition, there is no sharp absorbance peak detected in the M_11_ and S_22_ zones, which is different from previous reports [31,40]. We attribute this phenomenon to the existence of SWCNT bundles in the solution [41], the overlap of S_22_ and S_33_ [39], or the overlap of different chiral s-SWCNTs.

The enrichment of s-SWCNTs in the s-SWCNTs-rr-P3DDT sample was further characterized using laser Raman spectroscopy excited with 633 nm and 532 nm lasers. Figure 2c,d and Figure S8 show typical Raman spectra of s-SWCNTs-rr-P3DDT and r-SWCNTs-SDS samples, respectively. The r-SWCNTs-SDS sample (Figure S8) shows similar RBM Raman spectra with that of raw SWCNTs, suggesting that m- and s-SWCNTs coexist in the solution. On the contrary, very few RBM peaks excited from m-SWCNTs are observed for the s-SWCNTs-rr-P3DDT sample as shown in Figure 2c,d. Additionally, a relatively large amount of high density peaks at 145 cm^−1^ under 532 nm (2.33 eV) laser and 125 cm^−1^, 145 cm^−1^ under 633 nm (1.96 eV) laser, originating from semiconducting tubes with diameters ranging from 1.7 to 2.0 nm in s-SWCNTs/rr-P3DDT sample are detected. Therefore, both UV–Vis–NIR absorbance and Raman characterization show the same conclusion of the enrichment of s-SWCNTs with large diameters in the s-SWCNTs-rr-P3DDT sample, demonstrating the effectiveness of conjugated rr-P3DDT polymer in separating large-diameter s-SWCNTs.

**Figure 3:**
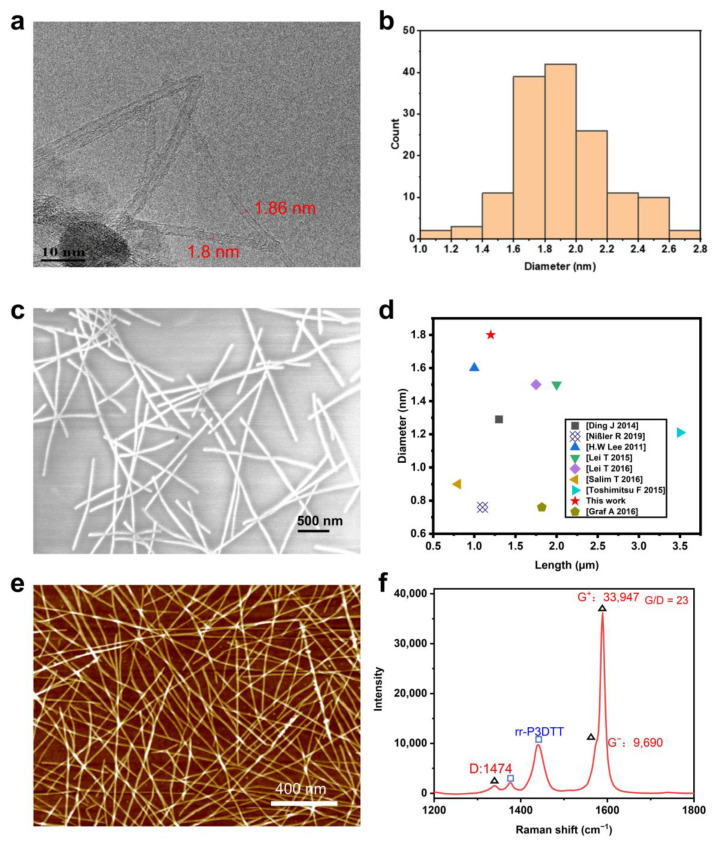
Characterization of s-SWCNTs/rr-P3DDT: (**a**) TEM image, (**b**) statistics of diameter distribution, (**c**) SEM image, (**d**) a comparison of tube diameter and length with previous reports [31,32,38,42,43,44,45,46], (**e**) AFM image, (**f**) G- and D-band Raman spectra under a 532 nm laser.

TEM observations (Figure 3a) show that the s-SWCNTs-rr-P3DDT obtained are straight and mostly isolated. The measured diameters of s-SWCNTs/rr-P3DDT under TEM are plotted in Figure 3b, which demonstrates that the diameters widely distribute and locate in 1.0~2.8 nm. The mean diameter of s-SWCNTs/rr-P3DDT is ~1.9 nm, which is much larger than results previously reported in the references [32,38,42,43,44]. SEM image shown in Figure 3c demonstrates that SWCNTs with lengths in micron scale are randomly distributed on the substrate, which is much longer than most previous reports [31,45,46]. The lengths of s-SWCNTs/rr-P3DDT were also measured from SEM images, and the obtained length distribution is shown in Figure 3d. The s-SWCNTs length distributed in the range of 0.5~3.2 µm, and 66% SWCNTs have a length over 1 µm. Compared with the results reported in typical references, our separated s-SWCNTs have the largest diameter and a relatively long length. Typical atomic force microscopy (AFM) image of Figure 3e further verified the long length of the s-SWCNTs/rr-P3DDT sample. At the same time, our s-SWCNTs show a high G/D ratio of 23 (Figure 3f), suggesting a low content of defects introduced during the separation process. The peak position of G^+^ band of the raw SWCNTs is at 1581 cm^−1^ and the peak position blue shift to 1589 cm^−1^ after the P3DDT sorting (Figure S9), indicating a p-type SWCNTs. Furthermore, the G^−^ band of the raw SWCNTs distributed in the range from 1558 to 1668 cm^−1^, while the G^−^ band of s-SWCNTs-P3DDT is located in 1572~1577 cm^−1^, exhibiting a Lorentzian line shape and further verifying the enrichment of s-SWNCTs. The intensity ratio of G^+^/G^−^ band of the raw SWCNTs is 2.09, and that of the s-SWCNTs-P3DDT is 3.50. The characteristics of the relatively large-diameter, longer length, and higher quality make the s-SWCNTs/rr-P3DDT sample suitable for the fabrication of transistors.

### 3.3. Fabrication of TFTs

**Figure 4:**
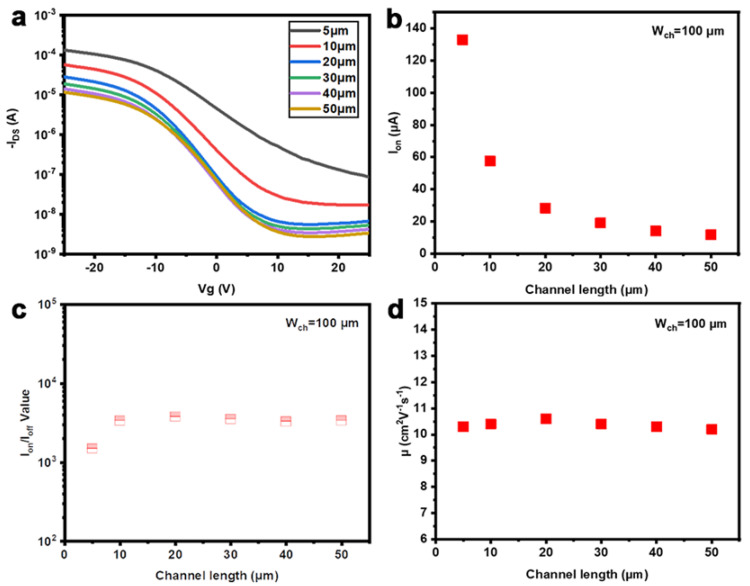
Electrical performances of the constructed TFTs with different *L*_ch_ and fixed *W*_ch_ of 100 μm. (**a**) Typical transfer characteristics at $\;V_{DS}=-1V$. Dependence of (**b**) on-current, (**c**) current on/off ratio, and (**d**) carrier mobility on *L*_ch_.

We therefore further constructed a bottom-gate TFT using highly doped p-Si as substrate, in which the 100 nm thick SiO_2_ layer was used as a gate dielectric. The drain electrodes (Ti/Au: 5/50 nm), source, and bottom-gate were prepared using a typical process, including photolithography, electron-beam evaporation, and lift-off. The solution of r-SWCNTs/rr-P3DDT was used for forming the active channels by dip-coating between the patterned S/D electrodes. To deposit a uniform CNT thin film, the substrate was coated with a monolayer of hexamethyldisilazane, and then the solution of r-SWCNTs/rr-P3DDT was dip-coated between the patterned S/D electrodes at room temperature. Finally, CNTs outside the channel area were removed by oxygen plasma. The electrical transfer properties of fabricated TFTs were analyzed with a common semiconductor analyzer (Agilent B1500A) together with a probe station (Cascade M150) under atmosphere. We first investigated the effect of channel lengths (*L*_ch_) (Figure S10 on the performance of the constructed TFT with a channel width (*W*_ch_) of 100 μm. The transfer characteristics of a series of TFTs with different *L*_ch_ were plotted in Figure 4a. Additionally, Figure 4b–d shows the *L*_ch_-dependent on-current (*I*_on_), current on/off ratio, and carrier mobility (*μ*). We can see that *I*_on_ is almost inversely proportional to *L*_ch_ (Figure 4a,b), while the current on/off ratio remains to be higher than 10^3^ with varied *L*_ch_ (Figure 4c), suggesting the high s-SWCNT content in the channel [47,48]. Specifically, a large on-current up to 130 µA was achieved in the TFT with a 5 μm channel length. In addition, the corresponding carrier mobilities under ambient condition, as calculated from the formula of *μ* = (*L*_ch_/*W*_ch_)(1/*C*)(1/*V*_DS_)(d*I*_D_/d*V*_GS_) used in the parallel plate model, where *C* is a constant, are all around $10\;{\mathrm{cm}}^{2}V^{-1}s^{-1}$ independent of *L*_ch_ (Figure 4d), indicating the good uniformity of our s-SWCNT films.

## 4. Conclusions

For the first time, s-SWCNTs with a large diameter of ~1.9 nm were separated using conjugated polymer rr-P3DDT with long side chain, which is the largest diameter s-SWCNTs obtained by post-synthesis separation. The s-SWCNT content is estimated to be higher than 97.5%, which demonstrates the effectiveness of long side chain rr-P3DDT polythiophenes in separating large diameter s-SWCNTs from their metallic counterparts due to their extensive π-conjugated structure. The s-SWCNTs obtained are clean, long, and less defective. TFTs fabricated using the separated s-SWCNTs as channel material show current on/off ratios of higher than 10^3^, and a mean carrier mobility of around $10\;{\mathrm{cm}}^{2}V^{-1}s^{-1}$. Meanwhile, the TFTs showed a large on-current up to 130 µA, which makes it promising for use in devices with a large start-up open circuit current.

## Data Availability

The data supporting the current study are available within the article and its supplemental information, and any additional requests for data will be fulfilled by the correspondence (cliu@imr.ac.cn (C.L.)) upon reasonable request.

## Supplementary Materials

The following supporting information can be downloaded at: https://www.mdpi.com/article/10.3390/nano13132001/s1, Figure S1. Schematic showing s-SWCNT separation process using the conjugated rr-P3DDT polymer wrapping method. Figure S2. XPS C 1s spectra of (a) untreated SWCNTs, and (b) treated SWCNTs with H_2_O_2_. Figure S3. Statistics of bundle diameter in the raw SWCNTs. Figure S4. Radial breathing mode (RBM) Raman spectra of SWCNTs excited with (a) 532 nm and (b) 785 nm lasers. Figure S5. Raman spectra of SWCNTs exited with 633 nm laser. Figure S6. Thermogravimetric curve of the untreated SWCNTs. Figure S7. The absorption spectra of SDS. Figure S8. RBM Raman spectra of R-SWCNTs-SDS sample excited with 532 nm and 633 nm lasers. Figure S9. Enlarged G band of Raman spectra of (a) the raw SWCNTs and (b) s-SWCNTs-P3DDT. Figure S10. Optical image of the TFTs with different channel lengths.
