# Supplementary material for: Enrichment of Large-Diameter Semiconducting Single-Walled Carbon Nanotubes by Conjugated Polymer-Assisted Separation
Source: Nanomaterials (Basel). 2023 Jul 4;13(13):2001. doi: 10.3390/nano13132001 (PMC10343552; doi:10.3390/nano13132001)
Supplement: Supplementary file 1 [file nanomaterials-13-02001-s001.zip › nanomaterials-2437430-supplementary.pdf]

Supplementary information

Piao Xie 1,†, Yun Sun 1,†, Chao Chen 1, Shu-Yu Guo 1, Yiming Zhao 1, Xinyu Jiao 1, Peng-Xiang Hou 1,*, Chang Liu 1,* and Hui-Ming Cheng 1,2

1 Shenyang National Laboratory for Materials Science, Institute of Metal Research, Chinese Academy of Sciences, Shenyang 110016, China; pxie17s@imr.ac.cn (P.X.); yunsun@imr.ac.cn (Y.S.);
chchen19s@imr.ac.cn (C.C.); syguo14s@imr.ac.cn (S.-Y.G.); ymzhao19b@imr.ac.cn (Y.Z.);
xyjiao18s@imr.ac.cn (X.J.); cheng@imr.ac.cn (H.-M.C.)

2 Faculty of Materials Science and Engineering/Institute of Technology for Carbon Neutrality, Shenzhen Institute of Advanced Technology, Chinese Academy of Sciences, Shenzhen 518055, China

* Correspondence: pxhou@imr.ac.cn (P.-X.H.); cliu@imr.ac.cn (C.L.)

† These authors contributed equally to this work.

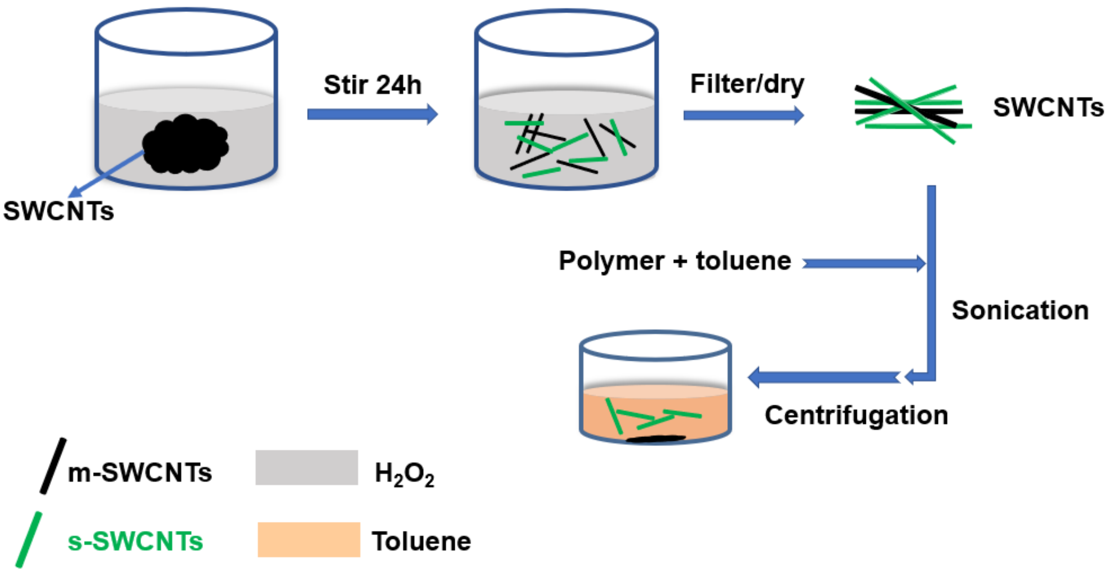

Figure S1. Schematic showing s-SWCNT separation process using the conjugated rr-P3DDT polymer wrapping method.

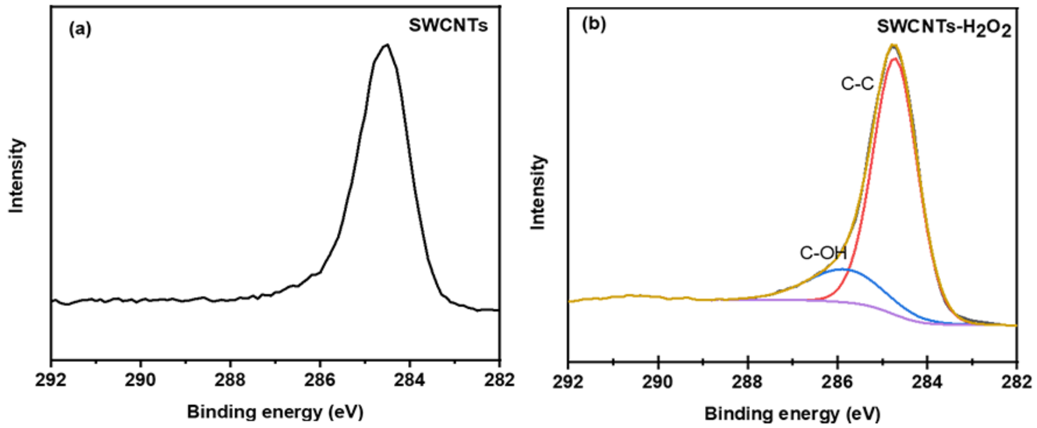

Figure S2. XPS C 1s spectra of (a) untreated SWCNTs, and (b) treated SWCNTs with H_2O_2.

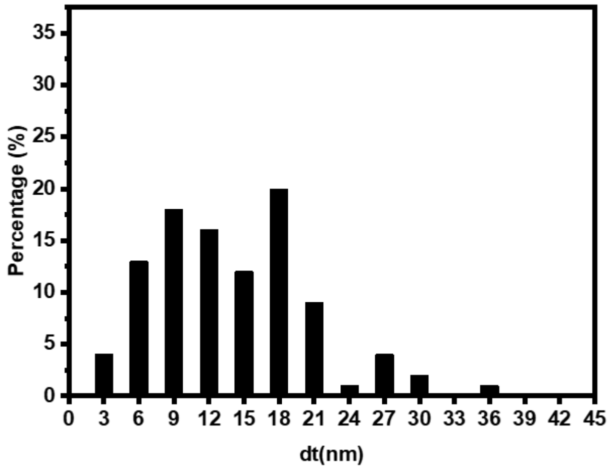

Figure S3. Statistics of bundle diameter in the raw SWCNTs.

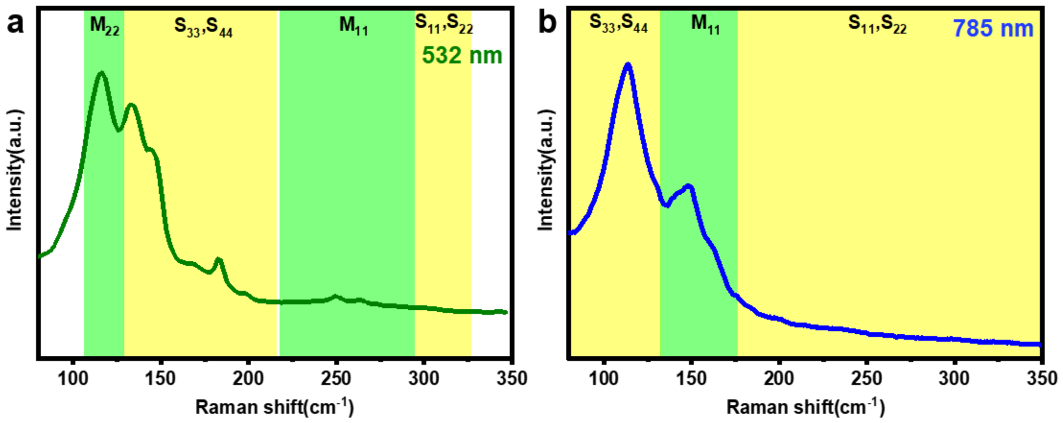

Figure S4. Radial breathing mode (RBM) Raman spectra of SWCNTs excited with (a) 532 nm and (b) 785 nm lasers.

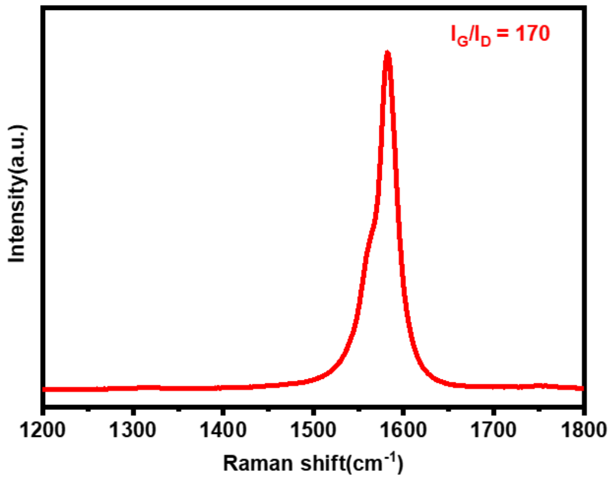

Figure S5. Raman spectra of SWCNTs exited with 633 nm laser.

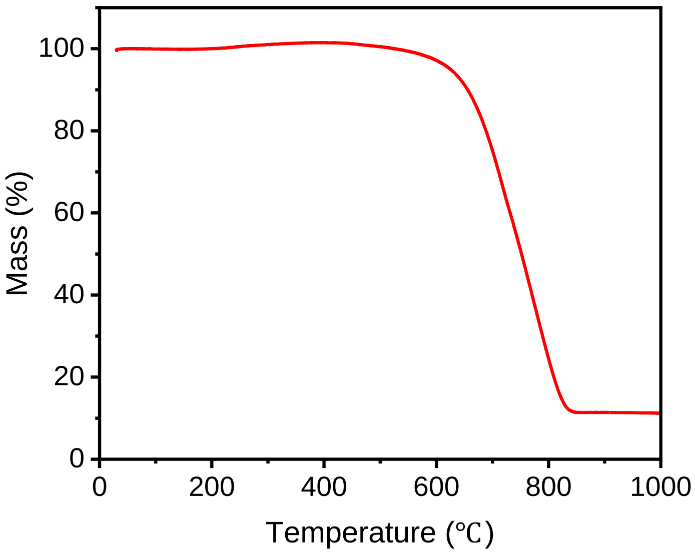

Figure S6. Thermogravimetric curve of the untreated SWCNTs.

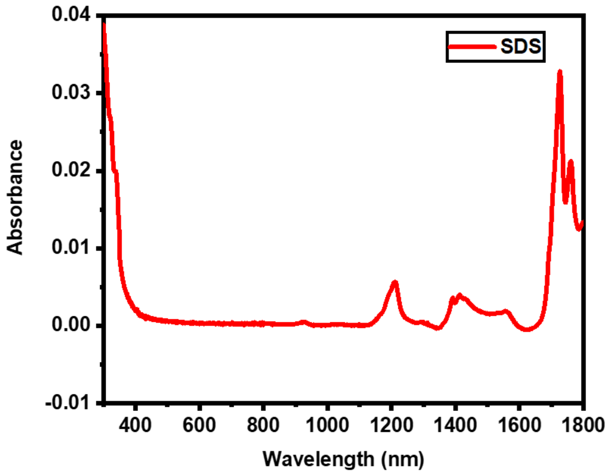

Figure S7. The absorption spectra of SDS.

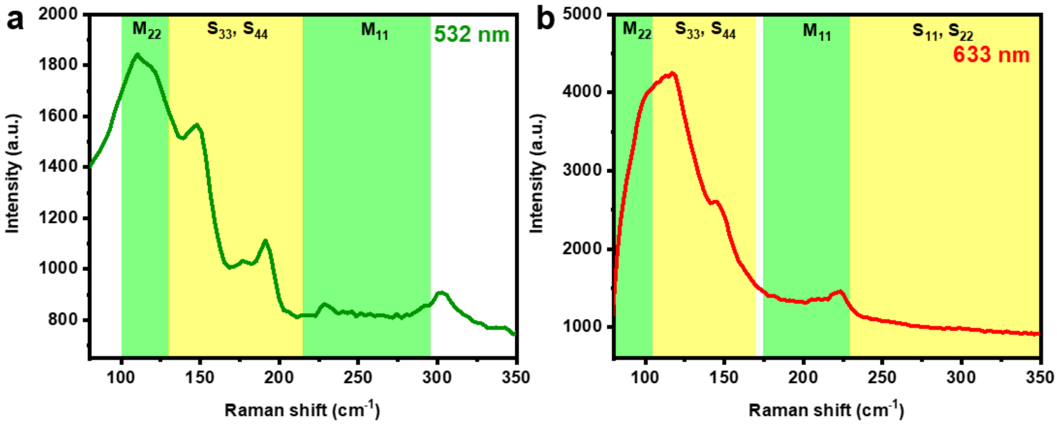

Figure S8. RBM Raman spectra of R-SWCNTs-SDS sample excited with 532 nm (a) and 633 nm (b) lasers.

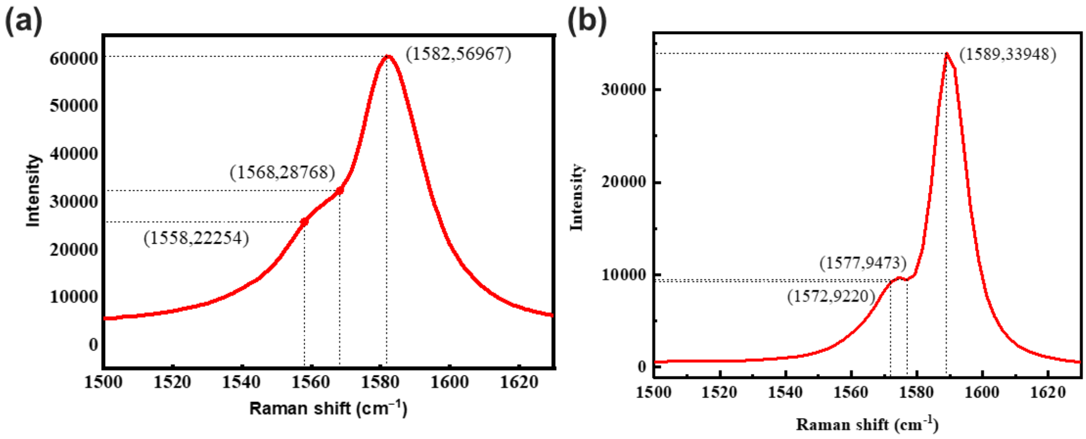

Figure S9. Enlarged G band of Raman spectra of (a) the raw SWCNTs and (b) s-SWCNTs-P3DDT.

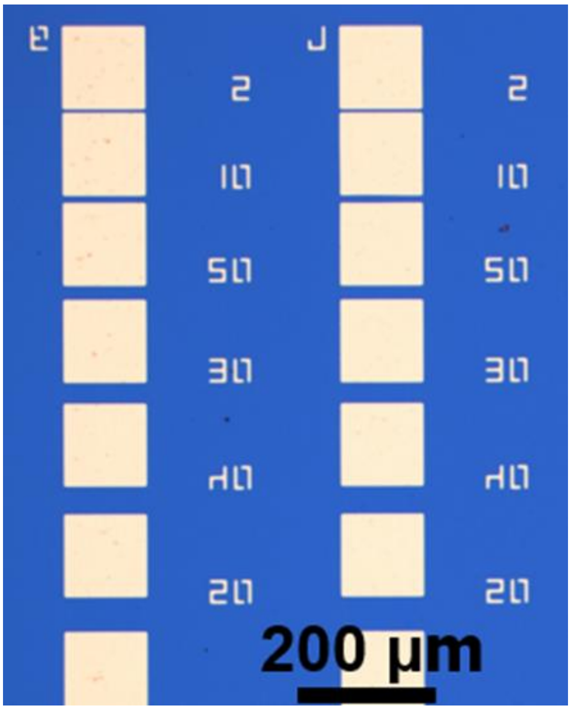

Figure S10. Optical image of the TFTs with different channel lengths.
